# Assessment of Insulin Injection Technique Among Diabetic Patients in Makkah Region in Saudi Arabia

**DOI:** 10.7759/cureus.10679

**Published:** 2020-09-27

**Authors:** Ghadeer A Alhazmi, Rafal N Balubaid, Samaher Sajiny, Rufaydah Alsabbah

**Affiliations:** 1 Medicine, Umm Al-Qura University, Makkah, SAU; 2 Internal Medicine, Umm Al-Qura University, Makkah, SAU; 3 Pediatrics, Umm Al-Qura University, Makkah, SAU

**Keywords:** diabetes mellitus type 1, diabetes mellitus type 2, insulin injection

## Abstract

Introduction: Diabetes mellitus (DM) is defined as a chronic metabolic disorder characterized by persistent high blood glucose. Proper insulin injection is indispensable to achieve adequate control of DM and prevent complications. Therefore, this study aims to assess the knowledge of DM patients about their insulin injection techniques.

Materials and Methods: A questionnaire-based and cross-sectional study was carried out from January to March 2020 at three locations in Makkah, Saudi Arabia. The inclusion criteria of research subjects included patients diagnosed with type-1 or type-2 DM and using insulin pen injection. The questionnaire included demographic data including age, gender, nationality, educational level, and chronic disease as well as specific questions about insulin injection technique.

Results: Four hundred and thirty-seven DM patients participated in the study. The most prevalent age group was between 20 and 60 years old (69.1%). The vast majority of patients were females (64.1%), Saudi nationals (92.9%), and residents of Makkah city (70%). There were roughly equal numbers of patients with type-1 and type-2 DM (47% and 53%, respectively). With regard to complications of DM, 19.5% of patients had previous acidosis, and 16.5% of patients were admitted to hospital for various complications. Injection-related complications were reported by almost half of the patients (49.9). The knowledge of insulin injection practices was examined among DM patients according to different variables. Only the frequency of administration exhibited a significant difference in the practices toward proper insulin injection technique (p = 0.049); patients who administered insulin injection three times daily had the best practices toward insulin injection when compared to other patients.

Conclusions: Our study showed that the practices toward insulin use among the surveyed DM patients in Makkah region were not satisfactory. Poor insulin injection technique is an important modifiable risk factor for uncontrolled blood glucose levels. More awareness campaigns and better counseling initiatives are crucial to guarantee steady insulin levels among DM patients.

## Introduction

Diabetes mellitus (DM) is defined as a chronic metabolic disorder characterized by persistent high blood glucose. It is one of the rapidly evolving disorders globally, and some countries have reached epidemic levels [1]. Specifically, Saudi Arabia is one of the leading ten countries worldwide with a high prevalence rate close to 24% [2].

Proper insulin injection and regular blood tests are indispensable to achieve an adequate control of DM [1,3-5]. Frid et al. demonstrated global improper insulin injection techniques by DM patients from 42 countries [6]. Contemporary reports have demonstrated a positive correlation between insulin injection misuse and poor glucose control [7,8].

Improper insulin injection technique is a common phenomenon. It can lead to glycemic variability and subsequently compromised short- and long-term complications [5,9]. Such aftermaths include injection site-related pain, bruising, allergy, infection, and lipodystrophy [10,11].

In Saudi Arabia, a recently published systematic review by Alanazi et al. depicted poor public awareness about DM and its risk factors as well as complications [12]. This poor awareness about DM raises a serious concern about the proper insulin injection techniques among DM patients. Therefore, the aim of this study is to assess the knowledge of DM patients about their insulin injection techniques.

## Materials and methods

A cross-sectional study using a questionnaire-based survey was carried out at three locations in Makkah, Saudi Arabia. These locations included: (1) primary healthcare centers, (2) the Department of Endocrinology at King Faisal Hospital Center, and (3) the Diabetic Center at Hira General Hospital. The study was conducted from January to March 2020. The inclusion criteria included patients diagnosed with type-1 or type-2 DM using insulin pen injection. Patients using insulin syringes were excluded. The research protocol was approved by the Ethics Committee at Umm Al-Qura University, Makkah, Saudi Arabia (identification number: HAPO-02-K-012-2018-08-08-263).

The questionnaire of study was prepared based on previous literature with modifications [13-15]. The questionnaire was conducted in an interview-based setting. There were two parts of the questionnaire with a total of 40 questions. The first part of questionnaire included questions about demographics, such as age, gender, nationality, educational level, and chronic diseases. The second part of questionnaire included questions relating specifically to DM, such as characteristics of DM, complications of DM, practices of insulin injection, insulin pen injection techniques, and sources of information about insulin injection.

The questionnaire was piloted on 20 research participants to gauge the validity and interpretation of questions. The questionnaire was found to be comprehensible, and thus no further changes were made.

With regard to questions about technical practices of insulin pen injection among the surveyed patients, each question which was answered correctly was given one point (a maximum score of nine points per patient).

Data were analyzed using the Statistical Package for the Social Sciences (SPSS, IBM Corp., Armonk, New York). Data were presented as numbers and frequencies. Two-tailed one-way analysis of variance (ANOVA) test was used to compare the average score of insulin injection techniques according to various sociodemographic and clinical variables. A p value less than 0.05 was regarded statistically significant.

## Results

Four hundred and thirty-seven DM patients participated in the study. Table 1 shows the patients’ sociodemographics. Age was subcategorized into three age groups, and the most prevalent age group was between 20 and 60 years old (69.1%). The vast majority of patients were females (64.1%), Saudi nationals (92.9%), and residents of Makkah city (70%). Close to half of the patients had a bachelor’s degree (49.9%) and other coexisting comorbidities (53.5%).

**Table 1:** The surveyed patients’ sociodemographics.

Variable		n (%)
Age	<20 years old	40 (9.2)
	20 to 60 years old	302 (69.1)
	60 years old	95 (21.7)
Gender	Male	157 (35.9)
	Female	280 (64.1)
Nationality	Saudi	406 (92.9)
	Non-Saudi	31 (7.1)
Educational level	Illiterate	36 (8.2)
	Primary	40 (9.2)
	Secondary	78 (17.8)
	Intermediate	30 (6.9)
	Bachelor	218 (49.9)
	Postgraduate	35 (8)
Existing comorbidities	Yes	253 (57.9)
	No	184 (42.1)

There were roughly equal numbers of patients with type-1 and type-2 DM (47% and 53%, respectively). With regard to complications of DM, 19.5% of patients had previous acidosis, 17.6% of patients had previous seizures, and 16.5% of patients were admitted to hospital for various complications.

Table 2 depicts information about the use of insulin by patients. Roughly 57% had been using insulin for more than five years. Insulin was self-administered in around three quarters of patients (72.1%) and mostly once (30.7%) or twice (30%) daily.

**Table 2:** Information about the surveyed patients’ use of insulin pen.

Question		n (%)
When did you start using insulin?	Less than two years	84 (19.2)
	Two to five years	104 (23.8)
	More than five years	249 (57)
What is the frequency of insulin administration?	Once daily	134 (30.7)
	Twice daily	131 (30)
	Three times daily	108 (24.7)
	Four times daily	55 (12.6)
	Five times daily	9 (2.1)
Who gives you insulin injection?	Someone	122 (27.9)
	Myself	315 (72.1)

Table 3 portrays the practices of insulin pen injection. Only 54.7%, 41%, 75%, and 63.2% of patients correctly had stored insulin inside fridge door, washed hands before injection, checked insulin expiration date, and used alcohol swab to rub the site of injection, respectively. Injection-related complications were reported by almost half of the patients (49.9%).

**Table 3:** The surveyed patients’ practices of insulin pen injection. * Correct answers for the questions.

Question		n (%)
Where do you store insulin?	Fridge door*	239 (54.7)
	Inside the fridge	164 (37.5)
	Room temperature	34 (7.8)
Do you wash your hands before injection?	Yes*	179 (41)
	No	104 (23.8)
	Sometimes	154 (35.2)
Do you check the expiry date?	Yes*	331 (75.7)
	No	60 (13.7)
	Sometimes	46 (10.5)
Do you check the type of insulin that you should take?	Yes	397 (90.8)
	No	40 (9.2)
What is the most common area that you inject?	Upper arm	151 (34.6)
	Thigh	148 (33.9)
	Abdomen	132 (30.2)
	Calf muscle	5 (1.1)
	Buttocks	1 (0.2)
Do you change the site of injection every time?	Yes*	241 (55.1)
	No	44 (10.1)
	Sometimes	152 (34.8)
What do you use to sterilize the injection site?	I do not rub injection site	128 (29.3)
	Water	4 (0.9)
	Medical swab*	276 (63.2)
	Tissue	29 (6.6)
Do you wait till the site of injection dries from alcohol?	Yes*	144 (33)
	No	187 (42.8)
	Sometimes	106 (24.3)
Do you inject the recommended dose?	Yes*	338 (77.3)
	No	51 (11.7)
	Sometimes	48 (11)
Do you notice any complications after using the needle?	Yes	48 (11)
	No	219 (50.1)
	Sometimes	170 (38.9)

Table 4 displays the technical practices of insulin injection. Only 65.7%, 9.6%, and 50.3% of patients correctly reported inserting the needle perpendicularly, discarding the needles in special baskets, and raising the skin at the site of injection, respectively. Around 65% of patients believed injecting insulin correctly.

About 58.7% had injection-related complications such as bleeding and bruising, while 35.3% of the patients reported lipohypertrophy.

**Table 4:** Technical practices of insulin pen injection among the surveyed patients.

Question		n (%)
What is the angle that you use to inject insulin?	Perpendicular (90 degrees)	287 (65.7)
	Inclined (45 degrees)	89 (20.4)
	Flat	61 (14)
Do you leave the needle in your body after injecting insulin?	Yes	245 (56.1)
	No	161 (36.8)
	I do not know	31 (7.1)
How long do you keep it?	Five to ten seconds	257 (58.8)
	Ten to 60 seconds	29 (6.6)
	More than 60 seconds	1 (0.2)
	I do not keep it	150 (34.3)
Do you rotate the injection site after the injection?	Yes	148 (33.9)
	No	166 (38)
	Sometimes	123 (28.1)
How many times do you reuse the needle?	One to five times	119 (27.2)
	More than five times	44 (10.1)
	I do not reuse it	274 (62.7)
Where do you throw the needle?	Special basket	42 (9.6)
	Bin	356 (81.5)
	Both	39 (8.9)
Do you return the cover to the needle after use?	Yes	399 (91.3)
	No	14 (3.2)
	Sometimes	24 (5.5)
Are you making skin fold?	Yes	220 (50.3)
	No	133 (30.4)
	Sometimes	84 (19.2)
Do you think that you inject insulin correctly?	Yes	283 (64.8)
	No	8 (1.8)
	Not sure	146 (33.4)

**Figure 1:**
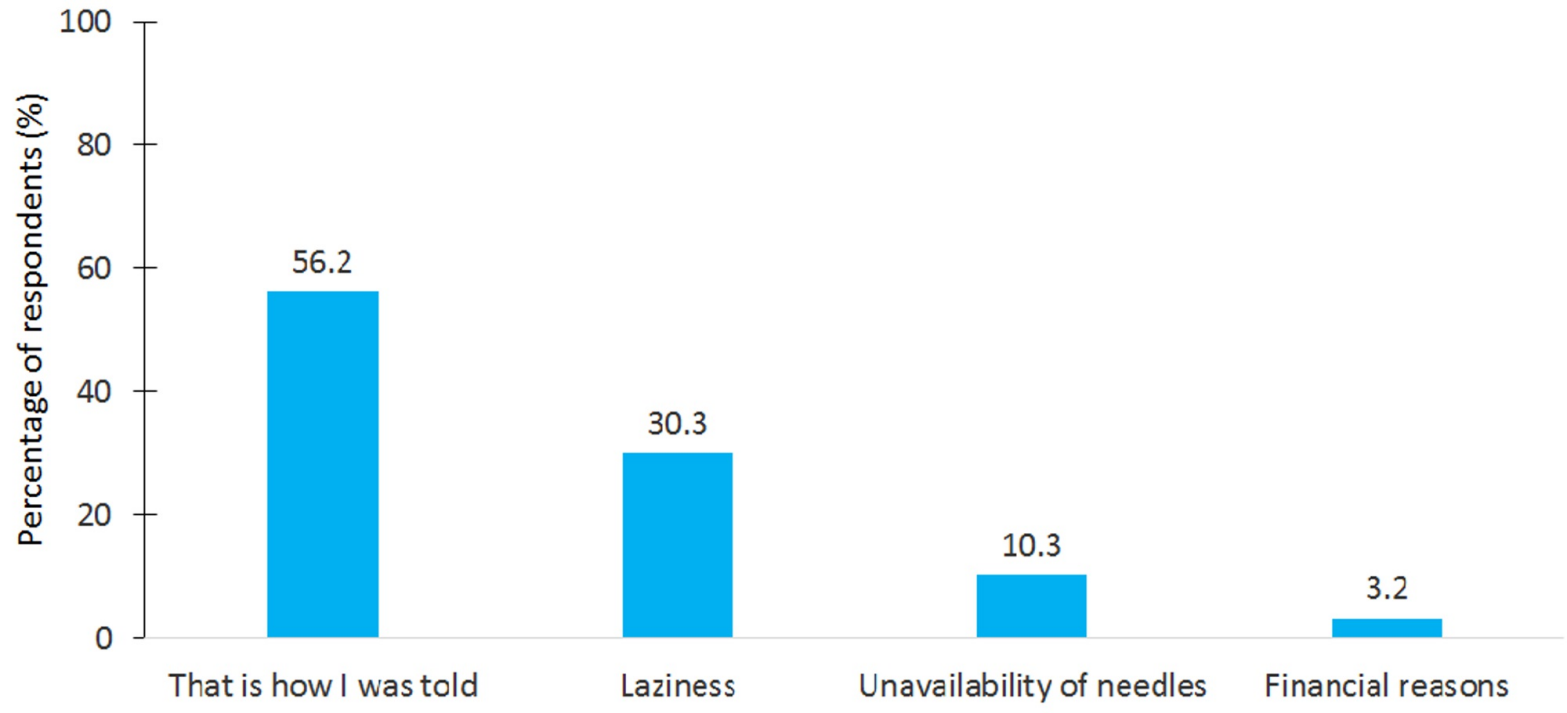
Reasons for reusing the insulin needle among the surveyed patients.

More than one third of patients (37.3%) reported reusing the needle more than one time. The reasons for reusing the needle are shown in Figure 1.

**Figure 2:**
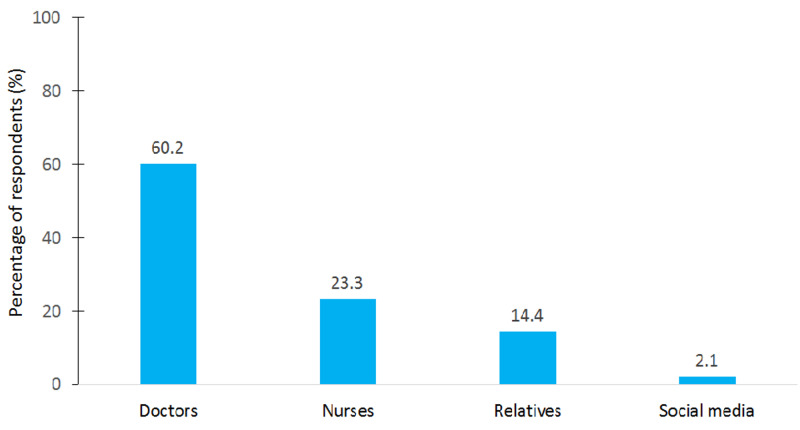
Sources of information about insulin injection among the surveyed patients.

The sources of information about insulin injection are depicted in Figure 2. The vast majority of patients obtained the information from doctors (60.2%), and only 2.1% of patients obtained them from social media.

Table 5 depicts the scores of the technical practices of insulin injection among the surveyed DM patients according to different sociodemographic and clinical variables. The mean ± standard deviation score of the entire cohort was 5.3 ± 1.8. Only the frequency of administration exhibited a significant difference in practices toward insulin injection (p = 0.049); patients who administered insulin injection three times daily had the best practices toward insulin injection when compared to other patients.

**Table 5:** Scores of insulin injection practices of the surveyed patients according to various variables. Two-tailed ANOVA test was used to compare the average score of insulin injection techniques according to various variables. A p value less than 0.05 was regarded statistically significant.

Variable		Mean ± standard deviation	p value
Age	<20 years old	5.0 ± 1.7	0.548
	20 to 60 years old	5.3 ± 1.9	
	>60 years old	5.3 ± 1.7	
Gender	Male	5.2 ± 1.7	0.353
	Female	5.3 ± 1.8	
Educational level	Illiterate	5.6 ± 1.8	0.436
	Primary	5.3 ± 1.8	
	Secondary	5.3 ± 1.7	
	Intermediate	5.5 ± 1.6	
	Bachelor	5.2 ± 1.8	
	Postgraduate	4.8 ± 1.8	
Type of diabetes	Type one	5.2 ± 1.7	0.635
	Type two	5.3 ± 1.8	
Duration of diabetes	Less than five years	5.5 ± 1.7	0.224
	Five to ten years	5 ± 1 .9	
	More than ten years	5.2 ± 1.7	
Type of insulin	Rapid-acting	5.2 ± 1.7	0.093
	Long-acting	4.1 ± 1.6	
	Mixed	5.5 ± 1.7	
	Long-acting and mixed	5.1 ± 2	
	Long-acting and rapid-acting	5.4 ± 1.8	
	Rapid-acting and mixed	5.0 ± 1.9	
	All of them	5.5 ± 1.6	
Frequency of insulin administration	Once daily	5 ± 1.8	0.049*
	Twice daily	5.2 ± 1.9	
	Three times daily	5.6 ± 1.4	
	Four times daily	5.5 ± 1.8	
	Five times daily	4.7 ± 1.9	
Do you think that you inject insulin correctly?	Yes	5.3 ± 1.9	0.490
	No	5.4 ± 1.7	
	Not sure	5.2 ± 1.8	
Do you notice any complications after using the needle?	Yes	5.3 ± 1.8	0.961
	No	5.2 ± 1.8	
	Sometimes	5.3 ± 1.7	

## Discussion

DM is a global health disease [13]. Hence, compliance of patients with their medical treatment is crucial to reduce the incidence of complications [13]. Insulin can be used as monotherapy or in combination with other therapies to control blood glucose levels. However, the maximal benefit of insulin depends on its appropriate administration [13]. Inappropriate insulin injection technique can result in erratic levels of insulin in the body, and it is one of the major reasons leading to poor glycemic control [11]. Accordingly, the incidence of DM complications can rise significantly leading to mortality [16]. Therefore, it is crucial to understand the practices of DM patients toward their insulin injection technique [14].

Saudi Arabia ranks among the top ten countries that harbor high prevalence rates of DM [2]. In this study, we sought to assess the practices of insulin pen use among DM patients in Makkah region, Saudi Arabia. In this study, we sought to assess the practices of insulin pen use among DM patients in Makkah region, Saudi Arabia. We targeted DM population about their insulin injection technique.

Our study showed that roughly one fifth of the surveyed patients experienced various DM-related complications, such as acidosis, seizures, and hospital admissions. Additionally, injection-related complications were reported by almost half of the patients. Moreover, our study showed that a large proportion of the surveyed patients had unsatisfactory knowledge about insulin injection practices. Overall, these data suggest the need to enhance DM control generally and improve the insulin injection practices specifically.

Knowledge about insulin use and its technique has been evaluated in different studies. Sweidan et al. examined the level of competency of insulin pen use among 165 patients in Saudi Arabia [15]. The authors showed that nearly 75% of the surveyed participants had poor knowledge. Poudel et al. scrutinized the insulin injection practices among 43 patients in Nepal [4]. The authors revealed that there were substantial gaps concerning proper insulin injection practice. Moreover, close to 30% of patients (n = 13) self-reported injection-related complications. Pozzuoli et al. demonstrated suboptimal insulin injection technique among 352 Italian patients with DM [17]. The authors reported a high (43%) prevalence of lipodystrophy among the studied subjects. A multivariate analysis revealed a positive association between occurrence of lipodystrophy and errors in insulin injection technique, such as improper rotation of needle at the site of injection and inappropriate spacing between insulin doses. Ji et al. conducted a national survey in China to gauge the daily insulin pen injection practice among 380 patients [18]. The authors demonstrated that close to one third of patients (35.3%) had lipohypertrophy, and more than half of the patients (58.7%) had injection-related complications such as bleeding and bruising. The authors highlighted practical errors in insulin pen injection among the surveyed DM patients.

Factors associated with reduced insulin adherence include smoking, young age, low socioeconomic status, level of scholarship, presence of other comorbidities, and polypharmacy [19]. In our study, only the frequency of administration exhibited a significant difference in practices toward insulin injection (p = 0.049); patients who administered insulin injection three times daily had the best practices toward insulin injection when compared to other patients. In line with an experience-based trend, we hypothesized that patients who inject insulin frequently would achieve the highest scores. The number of patients who used insulin five times and more was the smallest (n = 9), and this might have negatively affected the statistical analysis and masked the true outcome of an experience-based trend.

Our study mandates the need to boost the awareness of insulin injection technique among DM patients. The healthcare professionals-including physicians, nurses, and health educators-play the most central roles in this process. To that end, newly diagnosed patients with DM and requiring insulin injection should undergo one-to-one sessions about how to execute proper insulin injection. Moreover, those DM patients should be continuously counseled and examined for their insulin injection techniques during check-up visits. Lastly, educational campaigns and reliable social media platforms can provide reliable scientific materials to further strengthen the proper insulin injection practices.

The limitation of this study is that it depends mainly on patients' response to the questionnaire (recall bias) and not actual observation by healthcare professionals. As a result, the findings of this self-reported questionnaire are liable to overestimation/underestimation by the patients and may not accurately reflect the patients' actual responses. This limitation will be addressed in a forthcoming study in which patients will be observed by healthcare professionals and scored while injecting insulin.

## Conclusions

Our study showed that the practices toward insulin use among the surveyed DM patients in Makkah region were not satisfactory. Poor insulin injection technique is an important modifiable risk factor for uncontrolled blood glucose levels. Improving the use of insulin injection has favorable outcomes in reducing the risk of DM complications. More awareness campaigns and better counseling initiatives are crucial to guarantee steady insulin levels among the DM patients.
